# Derivation and characteristics of induced pluripotent stem cells from a patient with acute myelitis

**DOI:** 10.3389/fcell.2023.1172385

**Published:** 2023-07-14

**Authors:** Shuo Cao, Xinyue Gao, Fangyuan Liu, Yanglin Chen, Qin Na, Qiaoqiao Meng, Peng Shao, Chen Chen, Yongli Song, Baojiang Wu, Xihe Li, Siqin Bao

**Affiliations:** ^1^ The State Key Laboratory of Reproductive Regulation and Breeding of Grassland Livestock, Inner Mongolia University, Hohhot, China; ^2^ Research Center for Animal Genetic Resources of Mongolia Plateau, College of Life Sciences, Inner Mongolia University, Hohhot, China; ^3^ College of Basic Medicine, Inner Mongolia Medical University, Hohhot, China; ^4^ Inner Mongolia Saikexing Institute of Breeding and Reproductive Biotechnology in Domestic Animal, Hohhot, China

**Keywords:** induced pluripotent stem cells, acute myelitis, reprogramming, ectoderm, differentiation

## Abstract

The emergence and development of induced pluripotent stem cells (iPSCs) provides an approach to understand the regulatory mechanisms of cell pluripotency and demonstrates the great potential of iPSCs in disease modeling. Acute myelitis defines a group of inflammatory diseases that cause acute nerve damage in the spinal cord; however, its pathophysiology remains to be elusive. In this study, we derived skin fibroblasts from a patient with acute myelitis (P-HAF) and then reprogrammed P-HAF cells to iPSCs using eight exogenous factors (namely, *OCT4, SOX2, c-MYC, KLF4, NANOG, LIN28, RARG,* and *LRH1*). We performed transcriptomic analysis of the P-HAF and compared the biological characteristics of the iPSCs derived from the patient (P-iPSCs) with those derived from normal individuals in terms of pluripotency, transcriptomic characteristics, and differentiation ability toward the ectoderm. Compared to the control iPSCs, the P-iPSCs displayed similar features of pluripotency and comparable capability of ectoderm differentiation in the specified culture. However, when tested in the common medium, the P-iPSCs showed attenuated potential for ectoderm differentiation. The transcriptomic analysis revealed that pathways enriched in P-iPSCs included those involved in Wnt signaling. To this end, we treated iPSCs and P-iPSCs with the Wnt signaling pathway inhibitor IWR1 during the differentiation process and found that the expression of the ectoderm marker *Sox1* was increased significantly in P-iPSCs. This study provides a novel approach to investigating the pathogenesis of acute myelitis.

## Introduction

Human embryonic stem cells (hESCs) were first established by Thomson et al. (1998). Takahashi et al. (2007) derived the first human-induced pluripotent stem cells (hiPSCs). Human pluripotent stem cells (hPSCs), including hESCs and hiPSCs, have played an important role in regenerative medicine and disease research. hPSCs are characterized by the potential for self-renewal and multilineage differentiation and have since been established and studied in many laboratories (Loser et al., 2010; Fraga et al., 2011). Gao et al. (2019) established the expanded pluripotent human stem cell line (hEPSC) derived from iPSCs. Recently, Guo et al. (2021) found that hESCs differed from those of mice and showed that hESCs have the potential to differentiate directly into trophoblast cells.

iPSC technology has been extensively utilized for the development of cell therapies, drug discoveries, and disease modeling in humans. Researchers use differentiated cell populations derived from hESCs or hiPSCs to screen for effective compounds or new therapeutic targets affecting disease-related signaling pathways. Thus far, using iPSC technology, researchers have made significant progress in modeling diseases such as heart disease (Sasaki et al., 2016; McNally and Mestroni, 2017; Li et al., 2018), eye disease (Guo et al., 2018; Yan et al., 2019; Zhou et al., 2020; Klipfel et al., 2021), muscle disease (Choi et al., 2016; Ueki et al., 2017), and diabetes (Braverman-Gross et al., 2018; Yabe et al., 2019).

Acute myelitis, also known as acute transverse myelitis (ATM), is a rare inflammatory demyelinating disease. The incidence of ATM is estimated three cases per 100,000 population per year (0.003%). However, ATM can have devastating effects on the nervous system, with up to two-thirds of patients having moderate-to-severe disability (West et al., 2012). Symptoms of ATM include impaired sensory and autonomic function, which is easily recognized clinically. ATM is an acute or subacute condition with gradual deterioration within 4 h–21 days of onset (de Seze et al., 2005). ATM symptoms peak on average within 2–4 days, but not more than after 7 days, when urinary retention usually occurs. Thus, patients require catheterization, and in the acute phase, up to 89% of patients are unable to walk or need ventilator assistance for breathing (Kahn, 2020). Currently, acute inflammation in ATM is usually treated with high doses of intravenous corticosteroids, but also by plasmapheresis or intravenous immunoglobulin. Few studies have been conducted evaluating the pathogenesis of ATM.

Although iPSC disease models have played an important role in many studies, the current application of human stem cells in myelitis is mainly focused on hematopoietic stem cell transplantation for the treatment of optic neuromyelitis (Ceglie et al., 2020), and there have been no reports of the establishment of iPSCs in patients with acute myelitis. In this study, human embryonic fibroblasts and skin fibroblasts derived from a patient with acute myelitis were transfected with eight exogenous factors through the *PiggyBac* transposition method described by Gao et al. (2019) and reprogrammed into iPSCs. Here, we compared the biological characteristics of the patient iPSCs with normal iPSCs in terms of pluripotency, transcriptomic characteristics, and the ability to differentiate toward the ectoderm. This study provides an approach to explore the pathological mechanisms underlying acute myelitis.

## Materials and methods

### Materials

The human embryonic stem cell line W24 was obtained from the Wellcome Trust/Cancer Research UK Gurdon Institute. The normal human embryonic fibroblasts (HEFs) were received from Nanjing Medical University, and patient fibroblasts (P-HAF) derived from patients with acute myelitis were donated by volunteers at the hospital.

### Reprogramming human fibroblasts to iPSCs

A DNA mixture consisting of 3.0 μg PB-Tre-h4F, 1.0 μg PB-Tre-hRL, 1.0 μg PB-Tre-P2F, 1.0 μg EF1α, and 1.0 μg Pbase was prepared for transfection into cells. The transfected cells (1.0 × 10^6^) were seeded on STO (10-cm dish) in the M15 medium supplemented with doxycycline (Dox) (1.0 μg/mL). Dox was removed at day 14, and the medium was switched to the mTeSR (StemCell, 85,850) medium and expanded to stable iPSC lines.

### Culturing iPSCs/P-iPSCs

iPSCs/P-iPSCs were maintained on an STO feeder cultured in the M15+Dox medium, while iPSCs/P-iPSCs were maintained feeder-free and cultured in the mTeSR (STEMCELL, 85,850) medium. The colonies were dissociated by Versen for 3 min and removed of Versen, and then cells were suspended by mTeSR medium. Cell suspensions were placed a new well at a ratio of 1:5 and the mTeSR medium was replaced regularly every day. All culture plates used were coated with VTN (Gibco A14700) for at least 0.5 h prior to use.

### Alkaline phosphatase staining

Before staining, the cells were washed briefly with PBS and fixed in 4% paraformaldehyde at room temperature for 30 min, before adding the alkaline phosphatase (AP) staining solution. The AP staining solution was prepared as follows: 50 µL sodium nitrite solution was gently combined with 50 µL FRV alkaline solution, and the mixture was placed at 37°C for 3 min. Then, 2.25 mL of H_2_O and 50 µL naphthol-As-BI alkaline solution were added to the mixture.

### Karyotyping

A solution of 0.2 μg/mL colchicine was added to the cells in good growth conditions and treated at 37°C for 2.5 h. The cells were exposed to Versen for 3 min, and detached cells were centrifuged at 1,300 rpm for 3 min. The cells were placed in 0.075 mol/L KCL for hypotonic treatment for 50 min (stationary liquid: methanol: glacial acetic acid = 3:1). The cells were then centrifuged at 1,000 rpm for 10 min, and the cell pellets were placed in 8 mL of stationary medium at 37°C for 10 min. This step was repeated twice. Next, 300 μL ice-cold stationary liquid was added to the cell pellet and introduced dropwise onto glass slides. The glass slides were placed in a 70°C oven for 1 h. After drying, the cells were stained with Giemsa stain (Sigma) for 10 min at 37°C. Finally, the slides were placed under a microscope, and the chromosomes were arranged and analyzed using the LUCIA Cytogenetics platform.

### RT-qPCR

RNA was achieved using an RNA extraction kit (Qiagen, cat. no. 74104), and cDNA was prepared using Promega’s reverse transcription kit (cat. no. A5001). The primers used are listed in Supplementary Table S1.

### Immunofluorescence

Cells were washed briefly with PBS and fixed in 4% paraformaldehyde at room temperature for 30 min. Immunofluorescence (IF) buffer was added to permeabilize cells for 30 min at room temperature (0.1% Triton X-100 [Sigma] and 1% BSA in PBS). The primary antibody was prepared according to the antibody dilution ratio, and 150 μL of the primary antibody was added to each well before incubation at 4°C overnight. The next day, the cells were washed with IF buffer three times for 5 min each, and the secondary antibody was added at room temperature for 1 h in the dark. In addition, Hoechst 33,342 was added, and the nuclei were stained for 5 min at room temperature. The cells were analyzed using confocal microscopy. The antibodies used are listed in Supplementary Table S1.

### Bisulfite genome sequencing

Genomic DNA was extracted using the Genomic DNA Kit (TIANGEN Biotech, Beijing, China) and treated with the EZ DNA Methylation-Gold™ Kit (Zymo Research, Irvine, CA, United States). Then, the bisulfite-modified DNA was amplified by PCR using the following primers: Oct4 sense-GTTAGAGGTTAAGGTTAGTGGGTG and antisense-AAACCTTAAAAACTTAACCAAATCC. The amplified PCR products were randomly selected and cloned into the Pmd19T Cloning vector ((Takara, Otsu, Shiga, Japan) and sequenced.

### Differentiation of iPSCs/P-iPSCs

Pre-differentiated iPSC/P-iPSCs were detached using Versen and then seeded in 24-well plates at a density of 1.25 × 10^4^ cells in 1 mL of the M10 medium (DMEM/F12 supplemented with 10% FBS). After 12 days of culture, the cells were collected for analysis.

### RNA-seq and analysis

In this study, HEF cells and iPSCs were used as controls. Differential gene analysis was performed using the transcriptome data of HEF and P-HAF cells, as well as of iPSC and P-iPSC cells. Gene expression was first normalized, and differential analysis was conducted to select differentially expressed genes (DEGs) from the transcriptome sequencing results of HEF, P-HAF, iPSC and P-iPSC. Log2 Fold change >1 and *p*-value <0.05 were selected as screening criteria for differential genes. Gene ontology (GO) term enrichment analysis, Kyoto Encyclopedia of Genes and Genomes (KEGG) pathway enrichment analysis, principal component analysis (PCA), and Gene Set Enrichment Analysis (GSEA) were performed using OmicStudio and OmicShare tools, online platforms for data analysis. The PPI analysis was performed using String tools.

### Differentiation of hESCs, iPSCs, and P-iPSCs to the neural crest/cranial placode

The hESCs, iPSCs, and P-iPSCs were dissociated with 0.25% trypsin/EDTA and seeded in gelatin-coated well plates. Differentiation to neural crest (NC) cells: the cells were cultured in the BM medium supplemented with 1.0 ng/mL BMP4 (R&D systems, 314-BP-010), 10 μM SB431542 (Selleck, S1067), and 600 nM CHIR99021 (Miltenyi Biotech, 130-103-926). Two days later, the cells were cultured in the BM medium supplemented with 10 μM SB431542 and 1.5 μM CHIR99021. The cells were collected on day 12 for analysis. Differentiation to cranial placode (CP) cells: the cells were cultured in the BM medium supplemented with 5 ng/mL of BMP4 and 10 μM SB431542. Two days later, the cells were cultured in the BM medium supplemented with 10 μM SB431542 and 50 ng/mL bFGF (R&D systems, 233-FB-025). The cells were collected on day 12 for analysis. The BM medium (500 mL) was prepared as follows: 245 mL DMEM/F12 (Gibco, 11320-033), 245 mL Neurobasal Medium (Gibco, 21103-049), 2.5 mL N2 supplement (Gibco, 17502-048), 5.0 mL B27 supplement (Gibco, 17504-044), 5.0 mL 100 × GlutaMax (Gibco, 35050-061), and 0.1 mM 2-mercaptoethanol (Sigma, M3148).

### Western blot

Total protein was extracted from cells by cell lysis buffer (Solarbio, R1010) (PMSF and phosphatase inhibitor were added to lysis buffer immediately before use). The concentrations of extracted proteins were measured using the CoomassiePlus (Bradford) Assay (Thermo Scientific). Equal amounts of protein (30 μg) were separated by SDS-PAGE, and proteins were transferred to PVDF membranes (Amersham). The blots were blocked in 5% non-fat powdered milk in TBST at room temperature for 1 h and then incubated with the primary antibodies overnight at 4°C. The next day, secondary antibodies were incubated at room temperature for 1 h. The expression of protein was detected by ECL (Thermo Scientific).

## Results

### Establishment and global transcriptional features of HEF cells and P-HAF

ATM is a rare inflammatory demyelinating disease characterized by relatively acute motor, sensory, and autonomic dysfunction. ATM involves multiple components of the central nervous system, including oligodendrocytes, neurons, axons, and myelin, and is a mixed inflammatory disease rather than a pure demyelinating disease (Kerr and Ayetey, 2002; Krishnan et al., 2004; Weng et al., 2017b).

**FIGURE 1:**
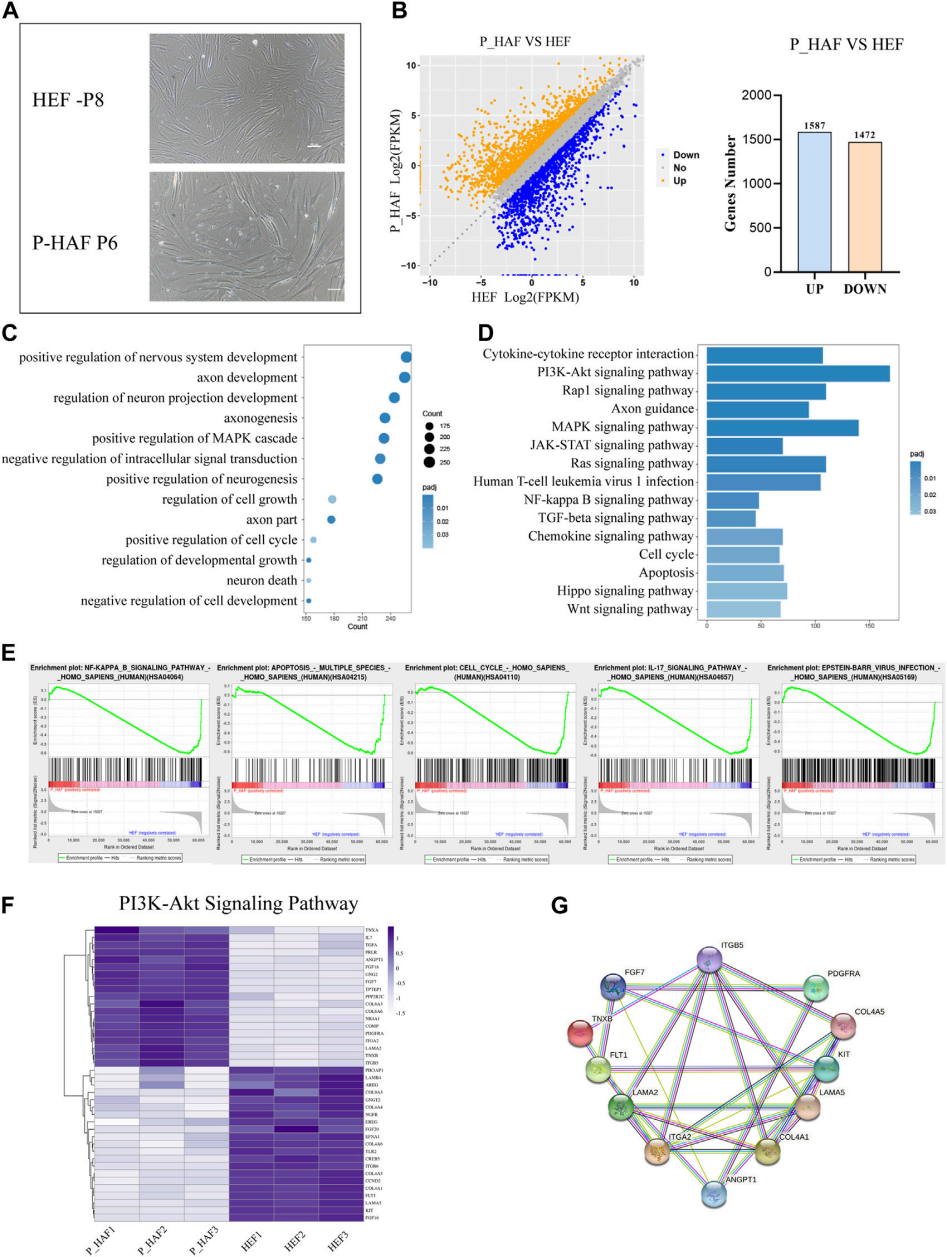
Transcriptomic features of HEF and P-HAF cells. **(A)** Morphology of HEF and P-HAF (scale bars, 100 μm). **(B)** DEGs analysis of HEF and P-HAF cells. **(C)** GO analysis of the DEGs between HEF and P-HAF. **(D)** KEGG pathway analysis of DEGs between HEF and P-HAF. **(E)** GSEA showing enriched GO-BP terms for upregulated DEGs and downregulated DEGs, respectively. **(F)** DEGs (|log2 (fold change)| > 1, FDR< 0.05) related to the PI3K-Akt signaling pathway are shown in the heatmap. **(G)** Network of candidate targets in the PI3K-Akt signaling pathway.

In order to establish iPSCs derived from the patient with ATM, we first established human embryonic fibroblasts (HEFs) and P-HAF derived from the patient. We show that HEF and P-HAF exhibited normal spindle-shaped symmetry (Figure 1A). To gain further insight into the underlying molecular mechanisms characterizing HEF and P-HAF, we performed RNA sequencing (RNA-seq) and analyzed DEGs using the DESeq2 R package. There were 3,059 DEGs in P-HAF compared to HEF cells, of which 1,472 genes were significantly downregulated and 1,587 genes were significantly upregulated (Figure 1B; Supplementary Table S2). To identify the functions of DEGs, we performed GO enrichment analysis. Comparing the GO enrichment results of HEF and P-HAF, we found that some terms involved in the positive regulation of nervous system development, axon development, and regulation of neuron projection development were enriched in DEGs (Figure 1C; Supplementary Table S3). KEGG pathway enrichment analysis revealed that enriched pathways were involved in the PI3K-AKT signaling pathway, the Hippo signaling pathway, the JAK-STAT signaling pathway, and the Wnt signaling pathway (Figure 1D; Supplementary Table S4). In addition, the GSEA showed enrichment in the NF-kB signaling pathway and in the IL-17 signaling pathway (Figure 1E). Furthermore, we observed that the expression of genes from the PI3K-AKT signaling pathway showed the most significant differences between HEF and P-HAF cells (Figure 1F). We used protein–protein interaction (PPI) to examine the top 12 proteins. The network revealed that ITGA2 and COL4A1 may play a key role in the PI3K-AKT signaling pathway (Figure 1G). Taken together, gene sets related to inflammation, Hippo, JAK-STAT, and Wnt signaling pathways are enriched in P-HAF cells.

### Establishment of iPSCs and P-iPSCs from HEF and P-HAF

**FIGURE 2:**
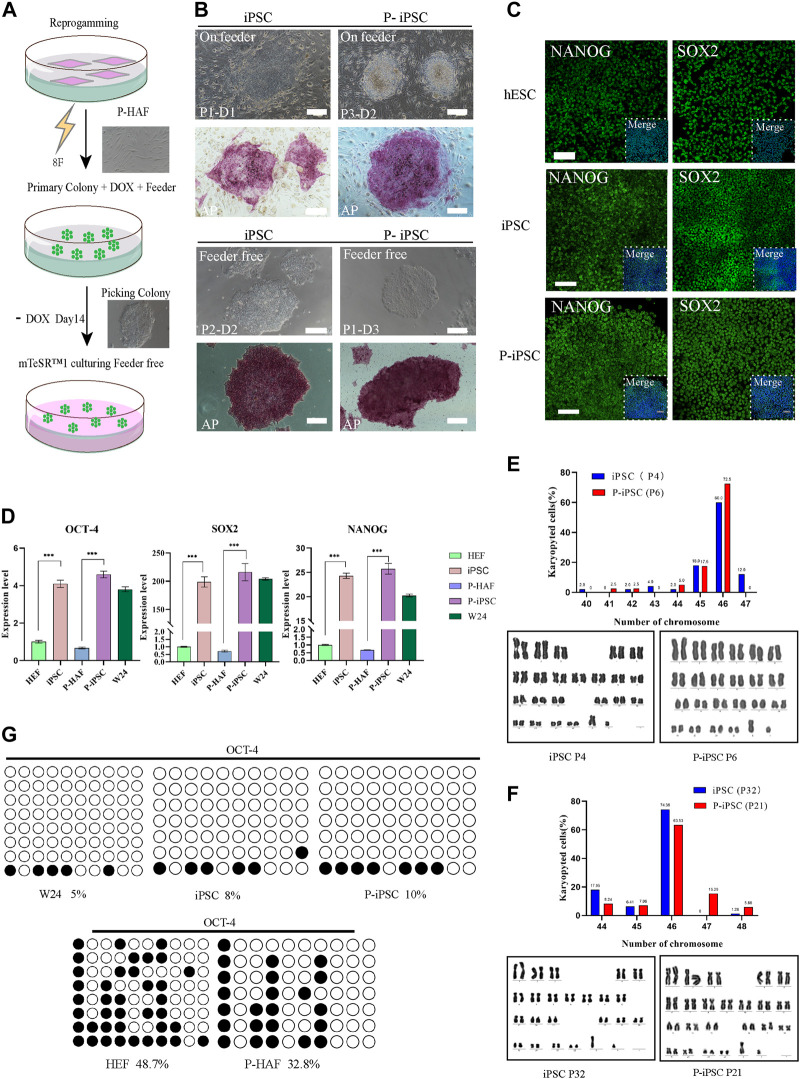
Reprogramming HEF and P-HAF cells to iPSCs, P-iPSCs, and the features of two types of iPSCs. **(A)** Schedule of reprogramming HEF and P-HAF to iPSCs and P-iPSCs. **(B)** Morphology and AP staining of iPSCs and P-iPSCs on feeder cells or feeder-free cultures (scale bars, 200 μm). **(C)** Immunostaining of NANOG and SOX2 in hESCs, iPSCs, and P-iPSCs (scale bars, 100 μm). **(D)** Real-time PCR analysis of pluripotency-associated gene expression in the hESCs, iPSCs, and P-iPSCs. HEF and P-HAF cells were used as the control. Data were obtained in triplicate and presented as the mean ± SD. *p*-values were calculated by two-way ANOVA, *p < 0.05, **p < 0.01, and ***p < 0.001. **(E)** Karyotyping analysis of iPSCs (passage 4) and P-iPSCs (passage 6). **(F)** Karyotyping analysis of iPSCs (passage 32) and P-iPSCs (passage 21). **(G)** Analysis of the DNA CpG methylation analysis of the *OCT4* promoter loci via bisulfite sequencing in HEF, P-HAF, hESCs, iPSCs, and P-iPSCs.

In this study, iPSCs were established by reprogramming normal HEFs and skin fibroblasts derived from a patient with ATM, using *piggyBac* transposition and doxycycline (Dox) inducible expression of eight exogenous factors (namely, *OCT4, SOX2, c-Myc, KLF4, NANOG, LIN28, RARG,* and *LRH1*) (Figure 2A). The Dox induction system allowed HEF and P-HAF cells to form primary colonies, which were selected from day 7 to day 14. The selected colonies were passaged in a serum-containing medium (MEM+15% FCS: M15) on STO feeder cells (Figure 2B). Based on the mechanical passaging method, the medium was changed to mTeSR, and the cells were passaged into cell culture dishes (the first passage was named P1), which were not feeder-or Dox-containing cultures (Figure 2B). We named the normal embryonic fibroblast-induced pluripotency stem cell as iPSCs and the patient (with ATM) skin fibroblast-derived iPSC cell line was named P-iPSC cells.

A remarkable feature of iPSCs and P-iPSCs was that they remained undifferentiated colonies under feeder-free conditions and without Dox for more than 30 passages, during which the cells proliferated robustly. Both iPSC and P-iPSC colonies were AP positive. Then, we determined the protein expression of SOX2 and NANOG, which were shown to be expressed at similar levels in hESCs (W24), iPSCs, and P-iPSCs (Figure 2C). The expression of pluripotency genes (*OCT4, SOX2,* and *NANOG*) in hESC (W24), iPSC, and P-iPSC was significantly higher than that of HEF and P-HAF cells (Figure 2D). Furthermore, iPSCs and P-iPSCs were genetically stable and exhibited a normal karyotype (Figures 2E, F). Furthermore, we found that the degree of DNA methylation of the *OCT4* promoter loci in HEF and P-HAF was higher than that in hESC, iPSC, and P-iPSC (Figure 2G), which is consistent with previous findings (Guan et al., 2022).

Taken together, we successfully established human iPSCs from normal embryonic and patient-derived fibroblasts using eight exogenous factors. We showed that the iPSCs and P-iPSCs display no obvious differences in the clonal morphology, both were positive for alkaline phosphatase activity and harbored normal karyotypes and activation of endogenous pluripotent gene expression in both lines.

### RNA-seq reveals iPSC and P-iPSC molecular features

**FIGURE 3:**
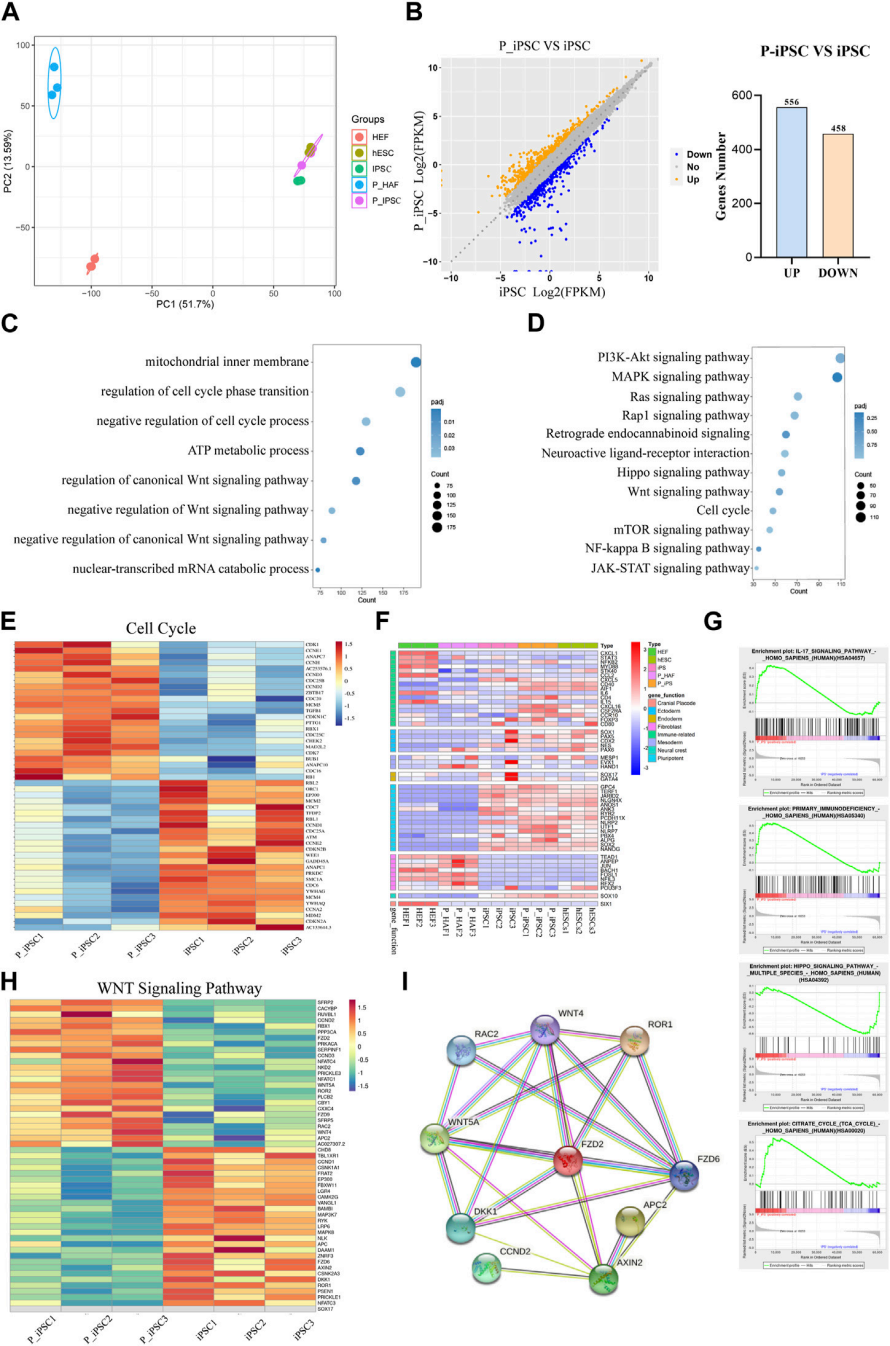
Transcriptome changes between iPSCs and P-iPSCs. **(A)** Principal component analysis of HEF, P-HAF, hESCs, iPSCs, and P-iPSCs. **(B)** DEGs of iPSCs and P-iPSCs. **(C)** GO analysis of the DEGs comparing iPSCs and P-iPSCs. **(D)** KEGG pathway analysis comparing iPSCs and P-iPSCs. **(E)** DEGs (|log2 (fold change)| > 1, FDR< 0.05) related to the cell cycle are shown in the heatmap. **(F)** Heatmap showing the expression of marker genes in HEF, P-HAF, iPSCs, P-iPSCs, and hESCs. **(G)** GSEA showing enriched GO-BP terms for up- and downregulated DEGs, respectively. **(H)** DEGs (|log2 (fold change)| > 1, FDR< 0.05) related to the Wnt signaling pathway are shown in the heatmap. **(I)** Network of candidate targets in the Wnt signaling pathway.

To gain further insight into the molecular mechanisms of P-iPSCs, we performed transcriptomic analyses by RNA sequencing of the HEF, P-HAF, hESCs, iPSCs, and P-iPSCs. PCA showed that iPSCs and P-iPSCs were closely related to hESCs and were distinct from HEF and P-HAF cells (Figure 3A). To examine the differences between iPSCs and P-iPSCs, we compared the DEGs. Overall, there were 1,014 DEGs in P-iPSC compared to normal iPSCs, of which 458 genes were significantly downregulated and 556 genes were significantly upregulated (Figure 3B; Supplementary Table S5). GO analysis revealed enrichment of the regulation of cell cycle phase transition, negative regulation of the Wnt signaling pathway, negative regulation of the canonical Wnt signaling pathway, mechanisms involving the mitochondrial inner membrane, metabolic processes involving ATP, and nuclear-transcribed catabolic processes of mRNA (Figure 3C; Supplementary Table S6). The KEGG pathway enrichment analysis showed the enriched pathways (Figure 3D; Supplementary Table S7), including the Hippo signaling pathway, the Wnt signaling pathway, the cell cycle, and the JAK-STAT signaling pathway. Notably, we observed that the expression of cell cycle genes was significantly different between normal iPSCs and P-iPSCs (Figure 3E). The expression of fibroblast-related genes and pluripotency-related genes in HEF, P-HAF, iPSCs, P-iPSCs, and hESCs were then analyzed. We found that fibroblast-related genes, such as *ANPEP, JUN, FOSL1,* and *RFX2*, are highly expressed in HEF and P-HAF but not expressed in iPSC, P-iPSC, and hESC (Figure 3F). Pluripotency-related genes, such as *GPC4, JARID2, SOX2,* and *NANOG*, were highly expressed in iPSCs, P-iPSCs, and hESCs but not in HEF and P-HAF (Figure 3F). Furthermore, the GSEA showed enrichment of the Hippo signaling pathway and the IL-17 signaling pathway (Figure 3G). In particular, we observed significant expression of the Wnt signaling pathway genes between normal iPSCs and P-iPSCs (Figure 3H). We constructed PPI networks to examine the top 10 proteins. The PPI network revealed that WNT5A and FZD2 may play a key role in the Wnt signaling pathway (Figure 3I).

These results indicate that iPSCs, P-iPSCs, and hESCs are pluripotent and display molecular features of pluripotency. In addition, our data indicate that P-iPSCs exhibit unique molecular features compared to iPSCs.

### Developmental potency of iPSCs and P-iPSCs

**FIGURE 4:**
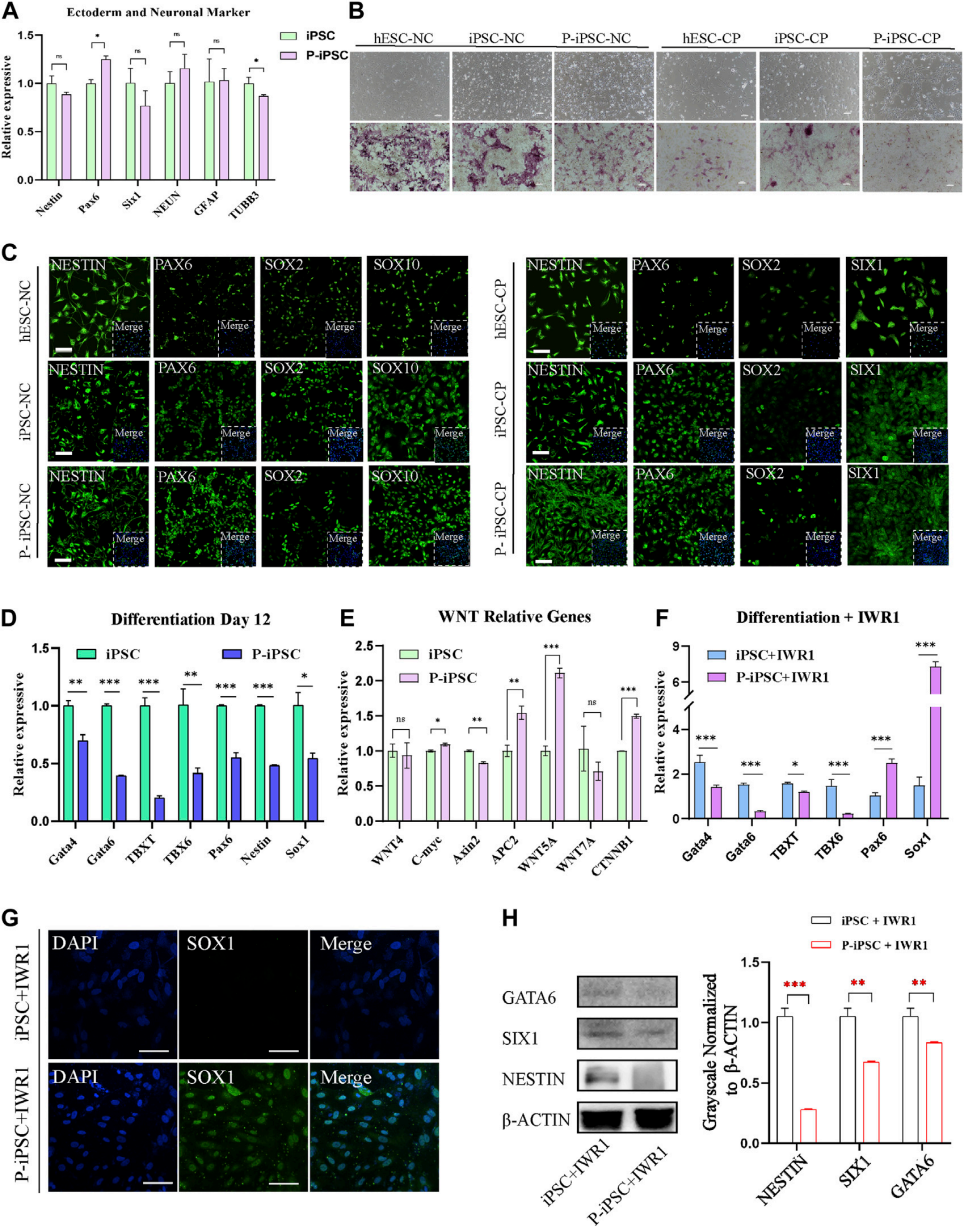
Differentiation of iPSCs and P-iPSCs toward the ectoderm. **(A)** Real-time PCR analysis of ectoderm- and neuronal-associated gene expression in the iPSCs and P-iPSCs; iPSCs were used as the control. Data were obtained in triplicate and presented as the mean ± SD. *p*-values were calculated by two-way ANOVA, *p < 0.05, **p < 0.01, and ***p < 0.001. **(B)** Cell morphology and AP staining of hESCs, iPSCs, and P-iPSCs differentiated to the ectoderm (scale bars, 200 μm). **(C)** Immunofluorescence for ectoderm-oriented cell markers derived from hESCs, iPSCs, and P-iPSCs (scale bars, 100 μm). **(D)** Real-time PCR analysis of endoderm-, mesoderm-, and ectoderm-related gene expression in iPSCs and P-iPSCs differentiated on day 12; iPSCs were used as the control. **(E)** Real-time PCR analysis of the Wnt signaling pathway-associated gene expression in the iPSCs and P-iPSCs; iPSCs were used as the control. **(F)** Real-time PCR analysis of endoderm-, mesoderm-, and ectoderm-related gene expression in iPSCs and P-iPSCs differentiated on day 12 of IWR1 treatment; iPSCs were used as the control. **(G)** Immunostaining of SOX1 in iPSCs and P-iPSCs differentiated on day 12 of IWR1 treatment (scale bars, 100 μm). **(H)** Western blotting analysis for GATA6, SIX1, and NESTIN in iPSCs and P-iPSCs differentiated on day 12 of IWR1 treatment, and the band intensity of Western blotting was quantified in the control.

To identify the differentiation property of P-iPSCs, we differentiated both P-iPSCs and iPSCs to the ectoderm. RT-qPCR analysis showed that the expression of ectoderm and neuron markers were significantly different between in iPSCs and P-iPSCs (Figure 4A). The ectoderm consists of four major ectoderm lineages, namely, the neuroectoderm, neural crest (NC), cranial placode (CP), and non-neural ectoderm (Tchieu et al., 2017). The neuroectoderm is the main structure that forms the central nervous system, and the NC and CP are involved in the formation of the peripheral nervous system (Breau and Schneider-Maunoury, 2014; Ji et al., 2019). To determine the capabilities of the iPSCs and P-iPSCs to differentiate into neurons, we used the human embryonic stem cell line (hESCs), iPSCs, and P-iPSCs to induce the formation of the NC and CP. According to the cell morphology and AP staining results, the NC and CP presented different cell morphologies, and the cell morphology of NC was similar to that of nerve cells, while the cellular morphology of CP resembled that of fibroblasts (Figure 4B). NC cells induced from hESCs, iPSCs, and P-iPSCs express the NC-specific marker SOX10 at the protein level and the ectodermal marker NESTIN and PAX6. CP cells induced from hESCs, iPSCs, and P-iPSCs express the CP-specific marker SIX1, as well as the ectoderm markers NESTIN and PAX6, while SOX2 is weakly expressed (Figure 4C). These data indicate that hESCs, iPSCs, and P-iPSCs were all capable of differentiating to NC and CP cells by our differentiation model, and these NC- and CP-induced cells showed comparable cell morphology and AP staining.

Interestingly, when we tested the ability of iPSCs and P-iPSCs to differentiate *in vitro* by the common method in the M10 basic medium for 12 days, we observed that the expression of all mesoderm, endoderm, and ectoderm marker genes was significantly decreased in P-iPSCs compared to iPSCs as analyzed by pPCR (Figure 4D).

Since our RNA-seq analysis showed enrichment of the Wnt signaling pathway in P-iPSCs when compared to iPSCs, we performed RT-qPCR on key genes, and the data were consistent with the RNA-Seq data (Figure 4E). We then treated iPSCs and P-iPSCs with 2 μM of the Wnt signaling pathway inhibitor IWR1 during the differentiation process and found that the expression of ectoderm markers *Pax6* and *Sox1* significantly increased in P-iPSCs (Figure 4F). These findings were confirmed by immunofluorescence staining against SOX1 (Figure 4G). Furthermore, the protein level of endoderm marker GATA6 and ectoderm markers SIX1 and NESTIN were downregulated in P-iPSCs compared with iPSCs differentiated on day 12 of IWR1 treatment (Figure 4H). These data indicated that after inhibiting the Wnt signaling pathway, the ectoderm marker *Sox1* was very sensitive in P-iPSCs. Previous studies have also shown that the Wnt signaling pathway plays an important role in the regulation of mESC (Ying et al., 2008) and in hESC pluripotency (Neagu et al., 2020; Bayerl et al., 2021). Recently, Liu et al. (2021) treated hPSCs with the Wnt activator (CHIR-99021) and leukemia inhibitory factor (LIF) in a chemically defined medium (N2B27) to induce neural stem cells (NSCs). Together, these results provide further support that the Wnt signaling pathway may play important roles in the differentiation of the ectoderm.

## Discussion

In this study, we successfully established human iPSCs from normal and patient fibroblasts. RNA sequencing data showed DEGs in iPSCs and P-iPSCs were enriched in the Wnt pathway, PI3K-AKT pathway, and JAK-STAT pathway. In particular, the NC and CP were successfully induced from iPSCs and P-iPSCs. Importantly, P-iPSCs were more sensitive to the Wnt pathway, and Wnt signaling inhibition resulted in an increased expression of SOX1, an ectoderm marker.

In 2008, iPSC technology was used to generate disease-specific iPSCs from somatic cells from patients with Parkinson’s, Huntington’s, and Gaucher’s disease (Park et al., 2008). Here, we used the *PiggyBac* eight-factor system and successfully established human iPSCs from the fibroblasts of normal individual and patient with ATM. A recent study also showed that using a similar eight-factor bovine protocol could establish expanded potential bovine stem cells (Zhao et al., 2021). iPSCs undergo epigenetic reprogramming (Takikawa et al., 2013; Li et al., 2019; Yagi et al., 2019), and some human iPSCs in culture displayed an increased chance of chromosomal abnormalities (Aasen et al., 2008). Using our approach, both iPSC and P-iPSC exhibited normal karyotypes (Figures 2E, F).

GSEA on HEF and P-HAF, as well as iPSCs and P-iPSCs, showed that the most significantly enriched pathways were related to inflammation. These findings indicate that although reprogramming will reduce the number of DEGs, some genes, such as those associated with inflammation-related pathways, are conserved in these processes. Thus, this process of establishing iPSCs using the eight-factor system is very stable. P-iPSCs can, therefore, be used as a model for further studies on ATM.

At present, research on ATM has focused more on clinical imaging analysis, treatment, and prognosis and is based generally on case reports. There are very few studies investigating the underlying pathologic mechanisms of ATM. In a study involving 42 patients with ATM, the levels of thyroid-stimulating hormone (TSH) and free triiodothyronine (FT3) in patients were lower than those in the healthy control group (Weng et al., 2017a). ATM, which involves multiple components of the central nervous system, including oligodendrocytes, neurons, axons, and the myelin sheath, is a mixed inflammatory disease (Kerr and Ayetey, 2002). In addition, histopathological studies have shown that ATM is associated with the activation of astrocytes and microglia (Krishnan et al., 2004).

The clinical features of ATM include a relatively acute onset of motor, sensory, and autonomic dysfunction, although peripheral nervous system lesions or damage have also been reported in patients with optic neuromyelitis and acute disseminated encephalomyelitis (Tavasoli and Tabrizi, 2018). To date, very little is known about the pathophysiology of the disease. Our study demonstrates that the *PiggyBac* eight-factor system is a successful approach for reprograming the patient fibroblasts to P-iPSCs, and it would be interesting to apply this in reprogramming thyrocytes, astrocytes, and microglia. These studies will provide powerful tools for understanding the pathogenesis of ATM.

Interestingly, we found that the expression of the ectoderm marker *Sox1* significantly increased in P-iPSCs following exposure to Wnt signaling inhibitor IWR1. The SOX1 and Wnt signaling pathways may be a clue to understand the underlying molecular developmental defects in ATM pathogenesis. Kan et al. (2004) reported that SOX1 binds to β-catenin, potentially attenuating the Wnt signaling pathway to regulate neurogenesis. Furthermore, Venere et al. (2012) found that SOX1 is expressed in a subset of astrocytes, and the subpopulation of SOX1-marked cells has long-term neurogenic potential. It remains to be investigated how SOX1 and Wnt signaling pathways play their roles in ectoderm differentiation.

## Data Availability

The datasets presented in this study can be found in online repositories. The names of the repository/repositories and accession number(s) can be found in the article/Supplementary Material.
