# Supplementary material for: Derivation and characteristics of induced pluripotent stem cells from a patient with acute myelitis
Source: Front Cell Dev Biol. 2023 Jul 14;11:1172385. doi: 10.3389/fcell.2023.1172385 (PMC10375497; doi:10.3389/fcell.2023.1172385)
Supplement: Supplementary file 6 [file Table1.DOC]

| **Reagent** | **Source** | **Identifier** | **Dilution Ratio** |
| --- | --- | --- | --- |
| **Antibodies for IF** | | |  |
| SOX2 antibody | R&D Systems | AF2018 | 1：200 |
| NANOG antibody | Ebioscience | 14-5761 | 1：500 |
| NESTIN | BOSTER | BM4494 | 1：200 |
| PAX6 | elabscience | E-AB-61653 | 1：200 |
| SOX1 | Bioss | bsm-54625R | 1：100 |
| SOX10 | Bioss | bs-20563R | 1：500 |
| SIX1 | Bioss | bs-17504R | 1：500 |
| Alexa Fluor 488 Donkey anti-Rabbit IgG（H+L） | life technologies | A-21206 | 1:500 |
| Alexa Fluor 488 Donkey anti-Goat IgG（H+L） | life technologies | A-11055 | 1:500 |
|  |  |  |  |

**Forward and reverse primers for qRT‐PCR.**

| **Genes** | **Primers** | **Sequences (5′‐3′)** |
| --- | --- | --- |
| GAPDH | Forward | CTCTGCTCCTCCTGTTCGAC |
|  | Reverse | TTAAAAGCAGCCCTGGTGAC |
| OCT4 | Forward | ATGCATTCAAACTGAGGTGCCTGC |
|  | Reverse | CCCTTTGTGTTCCCAATTCCTTCC |
| SOX2 | Forward | GCTGCAAAAGAGAACACCAATCCC |
|  | Reverse | AAACTTCCTGCAAAGCTCCTACCG |
| NANOG | Forward | GGTAGAAATTGGGGTTTAGAAAT |
|  | Reverse | TACAAAAAACAAACAACTTCCC |
| NESTIN | Forward | GCACCTCAAGATGTCCCTCAG |
|  | Reverse | CTGGGAGCAAAGATCCAAGAC |
| PAX6 | Forward | GTACTGAATGACTCAACTGCTCGG |
|  | Reverse | CTTTAGAAGGAAGCGACACTCTGC |
| SOX1 | Forward | CAACCAGGACCGGGTCAAACG |
|  | Reverse | GCCTCGGACATGACCTTCCACT |
| c‐MYC | Forward | CGACGAGACCTTCATCAAAAAC |
|  | Reverse | CTTCTCTGAGACGAGCTTGG |
| AXIN2 | Forward | CTCCGAGCTCACACTCAATTC |
|  | Reverse | GACAGGTGATCGTCCAGTATC |
| TUBB3 | Forward | GCCTGACAATTTCATCTTTGGTC |
|  | Reverse | CAGTCGCAGTTTTCACACTCCTT |
| NEUN | Forward | CCGAGTGATGACCAACAAGAAG |
|  | Reverse | CGCAGCCCGAAATGTATTATAC |
| GFAP | Forward | CACGCAGTATGAGGCAATGGC |
|  | Reverse | GGTAGTCGTTGGCTTCGTGCT |
| CTNNB1 | Forward | TGGATTGATTCGAAATCTTGCC |
|  | Reverse | GAACAAGCAACTGAACTAGTCG |
| GATA4 | Forward | CCTACCCGGCTTACATGG |
|  | Reverse | CTACGAGATTGGGGTGTCG |
| GATA6 | Forward | AGCACCAATCCCGAGAAC |
|  | Reverse | CGCCTATGTAGAGCCCATC |
| TBXT | Forward | CATGTGAAGCAGCAAGGC |
|  | Reverse | GGCTCTGGGGAAAGGTG |
| TBX6 | Forward | CATCCACGAGAATTGTACCCG |
|  | Reverse | AGCAATCCAGTTTAGGGGTGT |
| WNT4 | Forward | CCAGAGGCAGGTGCAGA |
|  | Reverse | AGACGGGCAAGGAGTCG |
| APC2 | Forward | GGACCGGGAACGGTGTTTC |
|  | Reverse | AGCTGAGAGTAGTACCAGAGC |
| WNT5A | Forward | GCCAGTATCAATTCCGACATCG |
|  | Reverse | TCACCGCGTATGTGAAGGC |
| WNT7A | Forward | TCGGGACTATGAACCGGAAA |
|  | Reverse | TGAGGAGAAGCCACCGATCC |
